# The Effects of Prehospital Care on Outcome in Pediatric Diabetic Ketoacidosis

**DOI:** 10.4274/jcrpe.galenos.2019.2019.0121

**Published:** 2020-06-03

**Authors:** Caner Turan, Ali Yurtseven, Elif Gökçe Basa, Damla Gökşen, Eylem Ulaş Saz

**Affiliations:** 1 Ege University Faculty of Medicine, Department of Pediatrics, Division of Pediatric Emergency, İzmir, Turkey; 2 Ege University Faculty of Medicine, Department of Pediatrics, İzmir, Turkey; 3 Ege University Faculty of Medicine, Department of Pediatrics, Division of Pediatric Endocrinology, İzmir, Turkey

**Keywords:** Diabetic ketoacidosis, prehospital care, diabetes mellitus, insulin, pediatric transport

## Abstract

**Objective:**

Despite the guidelines, significant variations can be encountered in initial therapy for pediatric diabetic ketoacidosis (DKA) in the prehospital setting. These variations occur mostly in fluid administration, insulin dosing, route of administration, and other aspects of the initial resuscitation and stabilization. The aim was to identify the effect of transport care on outcomes in children with DKA admitted to the emergency department (ED).

**Methods:**

Patients admitted to a tertiary-care pediatric ED between 2015-2019 with a diagnosis of DKA were retrospectively identified. Details of pre-pediatric ED care, including transport modality, patient demographics, clinical features, laboratory evaluation, fluid therapy, insulin dosing, and short-term outcome were recorded.

**Results:**

The study cohort included 147 episodes of DKA in 136 patients aged 9 months-21 years. Emergency Medical Service (EMS) transported only 37.4% of cases. EMS utilization rate was significantly higher (p=0.003) in severe cases, most of whom were >10 years (p=0.04). During transport 85% received intravenous fluid bolus. Use of fluids other than normal saline was significantly higher when transport time was >30 minutes (p=0.001). Acute kidney injury and cerebral edema developed in 21.7% and 7.4% of episodes, respectively. These complications were more common in the EMS transport group. Pediatric intensive care admission rate was also higher in the EMS compared to the non-EMS group (p=0.01).

**Conclusion:**

Parents did not call the ambulance for most cases although a higher complication rate occurred in EMS patients. EMS providers and referral facilities should improve their knowledge of pediatric DKA.

**What is already known on this topic?**

Despite the guidelines on initial management of pediatric diabetic ketoacidosis (DKA) significant variations (intravenous fluids and insulin therapy) can be observed in the prehospital setting or peripheral health care facilities.

**What this study adds?**

This is the first study exploring the low utilization rate of prehospital emergency medical services for children with DKA. Notably inappropriate fluid type/dose and insulin were administered in centers for primary/secondary care. Patients who received inappropriate initial management were more likely to develope complications.

## Introduction

Diabetic ketoacidosis (DKA) is one of the serious acute complications of type 1 diabetes mellitus (T1DM). DKA occurs at the onset of diabetes in half of patients (1,2). The annual rate of DKA in pediatric T1DM is 6-8%, with a case fatality rate ranging from 0.15% to 0.31% in developed countries. However, recent data from developing countries has shown that the mortality rate in children with DKA was 6-24% (3,4,5).

The principles of management of DKA in the pediatric population include optimization of: 1) volume status; 2) hyperglycemia and ketoacidosis; 3) electrolyte abnormalities; and 4) potential precipitating factors. Although, the management of these patients should be organized in comprehensively equipped or tertiary hospitals, the initial interventions performed before/during transport influence the final outcome. Thus some concerns remain about pre-hospital care of DKA in children (6). Despite the guidelines and recommendations concerning the optimal type and amount of intravenous (iv) fluid in the initial resuscitation of DKA, significant variations of initial iv fluid treatment occur in clinical practice. Similar inappropriate interventions including sub-optimal insulin dosing, route of administration, and other aspects of the initial resuscitation and stabilization, which are provided before transfer, have been reported (7).

This is, to the best of our knowledge, the first study exploring the utilization rate of prehospital Emergency Medical Services (EMS) for children with DKA, and the effect of transport modality-provided care and the association of these with prognosis. Interventions performed before referral and en route, administered fluid type and dose, insulin dosing and route of administration were also investigated.

## Methods

### Study Design

This is a retrospective cohort study conducted in the Emergency Department (ED) of Ege University Children’s Hospital between 1^st^ January 2015 and 31^st^ May 2019. The Ege University Local Ethical Committee (18-7/8) approved this study.

### Definition and Treatment Protocol

DKA was defined based on the International Society for Pediatric and Adolescent Diabetes (ISPAD) clinical practice consensus guidelines (8). According to this guideline, DKA is defined by the presence of all of the following; hyperglycemia (blood glucose >200 mg/dL), metabolic acidosis (venous pH <7.3 or serum bicarbonate <15 mEq/L) and ketosis (blood beta-hydroxybutyrate >3 mmol/L or moderate-large urine ketones). Our institutional management protocol for DKA was consistent with the ISPAD guidelines. The severity of DKA was structured into three groups: mild (pH=7.2-7.29), moderate (pH=7.1-7.19), and severe (pH <7.1).

For children with moderate and severe acidosis, initial resuscitation of 20 mL/kg of isotonic sodium chloride solution (0.9%), for mild cases and 10 mL/kg fluid was administered over 60 minutes. Following the initial fluid resuscitation, continuous, low-dose iv insulin infusion rate of 0.05 U/kg/hr was administered for children who were younger than 5 years old, and 0.1 U/kg/hr rate was used for children older than 5 years. Any exceptions to these recommendations for fluid administration (type/dosing) or insulin dosing during transport in EMS were defined as an inappropriate fluid or insulin therapy.

### Study Population and Data Collection

All patients admitted to our ED with DKA were included in the study. The data collection form included information on: transport modality (ambulance or not); patient demographics; clinical features; laboratory evaluation; administered resuscitation therapy (fluid type and volume, insulin type and dose) en route; and stabilization treatment in the ED. We also reviewed the medical records of all hospitalized patients to identify any subsequently developed complications, such as acute kidney injury (AKI) and/or cerebral edema (CE), and to collect outcome data during their hospital stay.

### Statistical Analysis

Statistics Package for the Social Sciences, version 22.0 software (IBM Inc., Chicago, IL, USA) was used for statistical analysis. Continuous data are represented by the mean and standard deviation or median and interquartile range (IQR), as appropriate. Categorical variables are expressed by frequency and cross tables. The chi-square test (or Fisher’s exact probability test) were used to compare demographics. Mann-Whitney U or t-test was performed for two independent groups, as appropriate. Values of p<0.05 were regarded as statistically significant.

## Results

**Figure 1:**
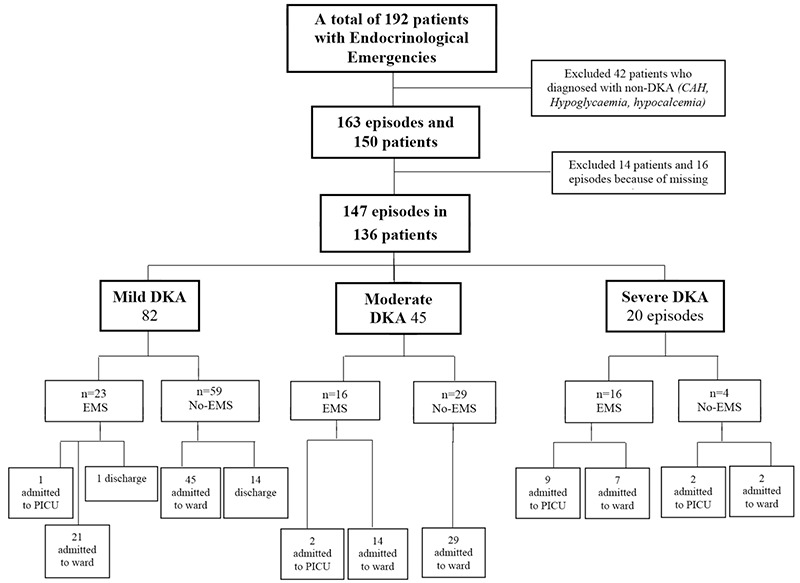
Distribution of episodes recruited in the study period DKA: diabetic ketoacidosis, CAH: congenital adrenal hyperplasia, PICU: pediatric ıntensive care unit, EMS: Emergency Medical Services

During the study period medical care was given to 192 endocrine emergencies in our ED. Most of them were DKA (163/192; 84.9%) involving 150 individual patients (Figure 1). We excluded 16 episodes in 14 patients due to missing data. The final analysis was performed for 147 episodes of DKA in 136 patients. Sixty-one percent were female and the mean age was 11.1±4.7 years (range 9 months to 21 years). Table 1 summarizes the demographic characteristics of patients in the study.

For most episodes, caregivers or parents did not choose to use ambulance transfer to the ED for their children (62.6%). EMS transported slightly more than one-third of this cohort (37.4%) (Table 1). The most common EMS transfers (43/55, 78.2%) were performed for patient referral from secondary care hospitals. EMS brought seven (12.7%) episodes from the field, and the remaining referrals were two (3.6%) from tertiary care ED and three (5.4%) from the primary care physician office. Patients who arrived at the ED without using EMS were more likely to have been sent from the primary care physician office (37.5%) (Table 1).

Tachycardia, dehydration, vomiting and altered mental status (Glasgow Coma scale ≤14) were more common in the EMS transported group when compared with the non-ambulance group. Comparisons of the clinical features in patients brought with or without EMS are shown in Table 1.

More than half of episodes (55.7%) were mild and only 13.6% were severe DKA (Figure 1). The comparison of patients’ laboratory findings between the severity groups of DKA are shown in Table 2. The mean pH was 6.90 (range was 6.70-7.07) in the severe group. EMS utilization rate was significantly higher in severe cases and most of the severe cases were adolescents (older than 10 years) (p<0.001, p=0.04 respectively) (Table 3).

Nearly half of the patients (42.8%) present with DKA at the time of diagnosis. The other most common causes of DKA presentation were; insulin omission (34.1%), insulin pump dysfunction (14.9%) and precipitating factors such as infections (8.2%). The proportion of children with new-onset T1DM and severe DKA was higher in the adolescent group. Although inappropriate fluid use was higher in patients under five years of age, complications were more common in patients older than five years (Table 4).

**Figure 2:**
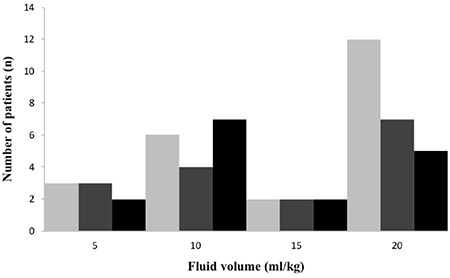
Administered intravenous fluid bolus volume (ml/kg) based on diabetic ketoacidosis severity

Most patients who were brought by EMS (45/55; 81.8%) received iv fluid bolus and the most common administered fluid (84.4%) was normal saline (0.9% NaCl) during the transport. Only a minority of episodes received inappropriate fluid type (such as 0.9% NaCl + 10% Dextrose, 0.45% NaCl + 10% dextrose or only 10% dextrose) (15.6%). Inappropriate fluid dosing was the most common mistake encountered (66.7%) (Figure 2). Inappropriate initial fluid doses and insulin treatments were associated with EMS transport (p<0.001 and p=0.009, respectively) (Table 3).

The EMS transfer group (39/55; 70.9%) arrived within one hour at the ED and only 16 patients’ transport duration lasted more than one hour. The rate of inappropriate fluid use was significantly higher when the transport time lasted more than 30 minutes (p=0.001).

In total 19 episodes received insulin therapy following fluid resuscitation. Although appropriate continuous, low-dose [begin with 0.05 (<5 years) to 0.1 U/kg/h], iv insulin infusion was performed for only six episodes, 13 episodes received inappropriate insulin therapy (11 subcutaneous; two subcutaneous and iv).

The median (IQR) length of stay in the ED was 2.1 (1.0-4.0) hours. AKI and CE developed in 21.7% and 7.4% of patients, respectively. These complications more likely to develop in moderate and severe DKA groups (Table 5). In addition, PICU admission rate was significantly higher in severe DKA who were transported by EMS, although there was no significant difference between EMS utilization and complications in severe DKA (p<0.001 and p=0.317, respectively) (Table 6). One hundred and seventeen episodes (80.2%) were admitted to the ward, and 14 to PICU. The rate of PICU admission was also higher in EMS when compared to the non-EMS group (p<0.001) (Figure 1). No significant association was found between the patients who received inappropriate interventions when compared with patients who received appropriate interventions during EMS with either PICU admission rate or complications.

## Discussion

The present study demonstrates several issues of concern regarding the prehospital management of pediatric DKA before referral and transfer to a tertiary care ED. Calling an ambulance (from the field to hospital) and using it for inter-hospital transport is the recommended response for patients with DKA in Turkey. There are several reasons for this. It is the most common emergency in pediatric T1DM and is still the major cause of hospitalization, morbidity, and mortality (1,2,9). Most morbidities and deaths due to DKA take place out of the hospital, presumably from severe dehydration and acidosis, which can be treated by iv fluids and insulin. If the primary goals of the management of DKA are performed appropriately and earlier in the clinical course, morbidity and mortality are clearly shown to be reduced (5). Unfortunately, this study shows that most children (62.6%) who have DKA do not access ambulances as their first medical contact. The reason for the low rate of EMS utilization is not clear and warrants further investigation. This is the first Turkish study to examine in detail the nature of the request for EMS in children with DKA. Since no previous study has published ambulance transport rates to hospital, our results suggest that patients preferred to use primary care physicians or secondary care hospitals and we believe that this may explain the higher rates of non‐ambulance transport observed in this study.

The incidence of DKA as the first presentation of new-onset T1DM has a large variability from country to country. Although, the DKA incidence of new-onset T1DM has been decreasing in European countries, such as Austria (34%), Germany (21.1%), Finland (22.4%), Denmark (17.9%), Italy (41.9%) and France (43.9%); this range was between 80-88% in African countries (1,10,11). In our country, this incidence was previously reported to be 33-55% (12,13). In the current cohort of patients,this rate was similar rate to the previous Turkish studies and to the reports from France and Italy. Precipitating factors such as infections, alcohol abuse, and insulin dose omission were the remaining main causes of DKA in diagnosed T1DM (14). Unlike in developed countries, where infection is the most common precipitating factor for DKA, insulin disruption/omission was the major precipitating factor for DKA in the studied patients (34.8%) (14).

The severity of the episodes in the previous studies was reported as mild/severe in 33% and 9%, respectively (13,15). In the current cohort, the severe DKA rate was 14.6% which is similar to data from Germany (16%), France (14.8%) and Italy (11.2%) and less than reports from Poland (22.5%) and Saudi Arabia (26.1%). This difference may be explained by lower parental educational achievement.

In previous studies, a young age, especially less than two years, and low accessibility to medical care were identified as risk factors for DKA at T1DM diagnosis (11,15,16,17,18). This association of young age and DKA at diagnosis may be explained by worse beta cell dysfunction, more aggressive diabetes and delayed detection of diabetic symptoms occurring more frequently in young children. It has been shown that children less than five years of age are at higher risk of metabolic decompensation at initial presentation (19). However, some studies indicated that informing the parents about diabetes symptoms decreased the risk of DKA at T1DM diagnosis in young children (20). In contrast to these studies, the present study found more frequent and more serious DKA episodes in children aged >10 years old. Children and adolescents at this age have likely escaped parental control. Thus, detection or reporting of symptoms may be delayed. Similar to our findings, a recent study from New Zealand reported an increased risk for DKA at age around 11 years (21). This may depend on better awareness in parents who have children <5 years and that adolescents do not recognize symptoms.

The management of DKA in any setting, both for patients with newly or previously diagnosed T1DM, can be divided into four physiologic principles which are restoration of fluid volume, inhibition of lipolysis, correction of electrolyte abnormalities and correction of acidosis. Delayed, insufficient or inappropriate treatment is a potential risk for developing complications of DKA (22). The timing of fluid therapy as an initial treatment has a considerable effect on the outcome of DKA and it is recommended that it should be given within the first hour (23). Since the majority of patients spend the first time when treatment of DKA could be given in the ambulance, if they use it, they should receive the initial therapy en route. Despite all the guidelines and recommendations this study has highlighted several concerns about the prehospital management of DKA (6). Although, only one-third of these cohort patients were brought by ambulance to the ED, there was still inappropriate fluid dose and insulin used for DKA during ambulance transport. The incidence of severe DKA and complications of kidney injury, CE and PICU admission rate were significantly higher in patients transported with EMS. The high rate of complications and morbidity associated with DKA in EMS-transported patients was related to both transport facilities and the severity of DKA. We believe that the majority of referring physicians from secondary care hospitals and prehospital healthcare providers attending to these children lacked the clinical experience to manage DKA. The uncorrected hypovolemia in our cohort may have resulted in complications. Since most severe patients were in the EMS group this would also explain the difference in the rate of developing complications. Since our study was conducted retrospectively and the sample size was small, further, well-designed prospective studies with a large sample size would be needed to clarify the situation.

There are many factors that contributed to the development of complications in the non-EMS group. Since the most common referral place in this group was the home, parents of children with T1DM should be advised to use ambulance transport when bringing their children with DKA to the ED. The second most common referral center in the non-EMS group was primary care and we believe that feedback should be made to primary care physicians for transferring these patients by EMS with an appropriate management protocol.

### Study Limitations

There are several limitations to the present study. The retrospective design of our study is the most crucial limitation which may have led to selection bias. In addition the single-center experience with a small sample size cohort were also limitations. Well-designed larger, prospective, multi-center studies are needed to explore and explain this causal relationship.

## Conclusion

The severity, complications, PICU admission and morbidity associated with DKA in our study was higher than that reported from developed countries. The root causes for the above were lack of parental education concerning DKA, inappropriate transport type and inappropriate therapy with fluid and/or insulin, and delayed management due to lack of clinical experience and facilities for managing DKA in the primary/secondary health-care facilities. This is compounded by transport problems associated with referral hospitals and lack of follow-up and continuum of care among known diabetics.

## Figures and Tables

**Table 1:**
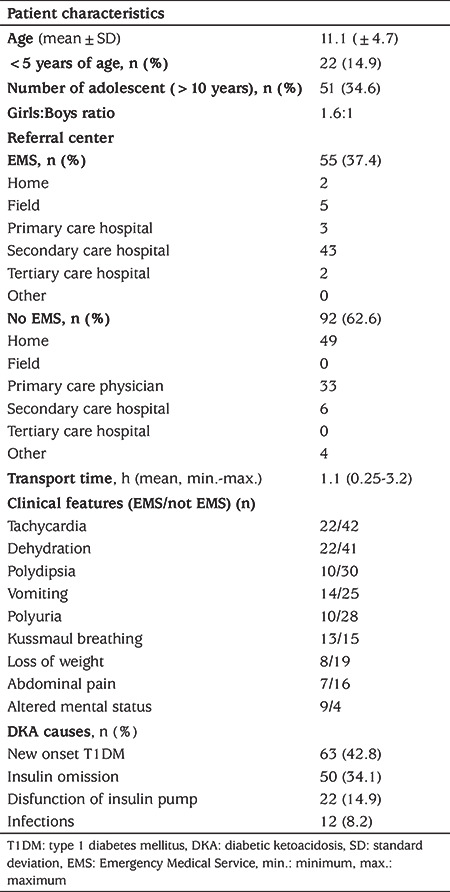
The patient characteristics and clinical features of episodes

**Table 2:**
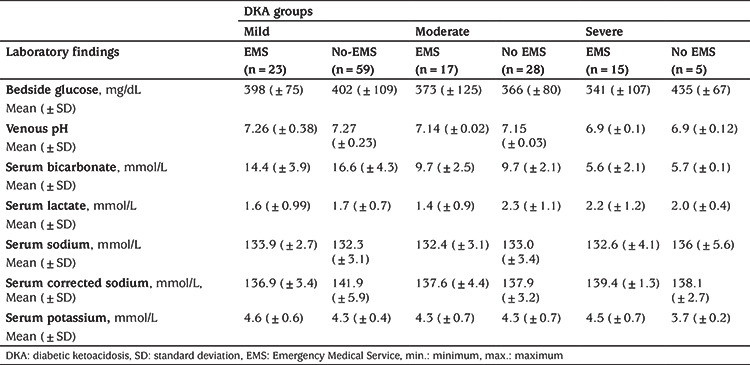
Comparison of patients’ laboratory findings between the severity groups of diabetic ketoacidosis

**Table 3:**
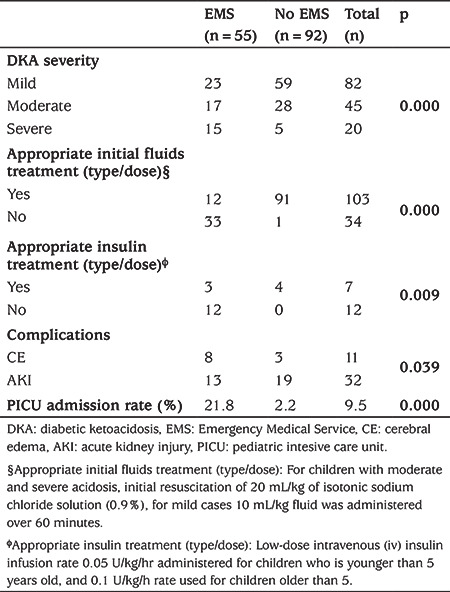
The association between diabetic ketoacidosis severity, appropriate treatments (fluid and insulin), complications, Pediatric Intesive Care Unit admission rates with Emergency Medical Service utilization

**Table 4:**
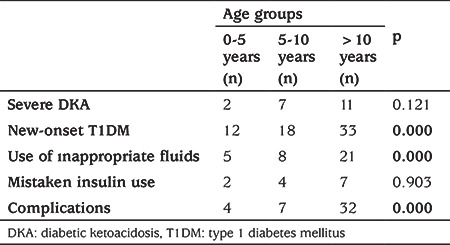
Comparison of clinical features, interventions and complications between the age groups

**Table 5:**
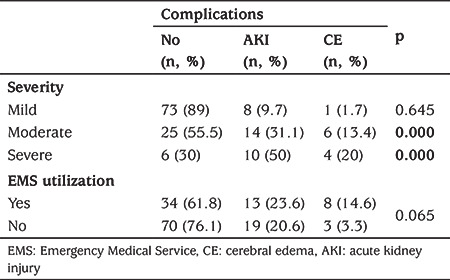
The association of diabetic ketoacidosis severity and Emergency Medical Service utilization between the rate of complications

**Table 6:**
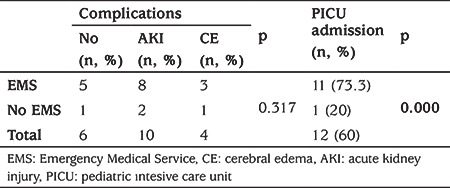
The comparison between the rate of complications, pediatric intesive care unit admission and transport modality in severe diabetic ketoacidosis group
